# Need for MRI scans in a real–world CIED population over long-term follow-up: Data from a large single-centre experience

**DOI:** 10.1371/journal.pone.0244672

**Published:** 2020-12-30

**Authors:** Giosuè Mascioli, Elena Lucca, Federica Michelotti, Luca Tarantino, Fabrizio Giofré, Ilaria Finamora

**Affiliations:** Division of Electrophisiology, Humanitas Gavazzeni Hospital, Bergamo, Italy; King's College London, UNITED KINGDOM

## Introduction

Magnetic resonance imaging (MRI) has become the first-choice imaging examination for many neurological, oncological and osteo-articular diseases. Its use also continues to grow in cardiac diseases [1]. Meanwhile, the number of patients implanted with a cardiac implantable electronic device (CIED) has also increased [2], making the possibility of a CIED-implanted patient needing an MRI scan more likely today than in the past.

In 2005, Kalin and coll [3].–citing data coming from a large CIED manufacturer–estimated that 50%– 75% of CIED-implanted patients could need an MRI scan during follow-up. For this reason, CIED manufacturers have invested in producing “MRI conditional” devices. The term “MRI conditional” identifies devices for which an MRI scan is safe–under a particular set of conditions. Apart from the question of cost, there are other factors to consider. For instance, the implantation of an MRI conditional device does not ensure MRI conditionality in the presence of old or abandoned leads. In addition, MRI conditionality is system-dependent, which requires all MRI conditional components to come from the same manufacturer; during a replacement intervention it is not always possible to ensure this.

In the absence of diagnostic alternatives, a European expert consensus considers the possibility of removing old and abandoned leads, after appropriate risk assessment, to allow an MRI scan [4]. In the US, lead removal may be considered for patients to facilitate access to MRI, but this a weak (class IIb) recommendation based on low quality evidence (expert opinion) [5]. In fact, several studies, involving a high number of patients [6], have demonstrated that MRI scans can be safely performed even in patients with non-MRI conditional devices. In CIED-implanted patients requiring MRI for the clinical management of serious diseases, European Society of Cardiology guidelines advocate an MRI risk-benefit assessment [7].

Nevertheless, since the study by Kalin and coll. published in 2005 [3], not many studies have investigated the real need for MRI scans in an unselected population of CIED patients. The aim of our study was to verify how many requests for an MRI scan had been received post-implant by regularly followed–up CIED-implanted patients from our centre.

## Material and methods

All consecutive patients who came to our outpatients clinic from December 2018 to October 2019 were questioned to find out: (i) if an MRI scan had been requested for them, (ii) if the exam had been performed, and (iii) which body zones had been studied. Patients unable to give reliable answers or patients who were unable to respond themselves who were not accompanied by a relative able to do so on their behalf were excluded from analysis. A verbal informed consent was obtained from all the patients enrolled in the study. It was witnessed by the patient himself, the physician who take care of the device follow-up and by the nurse who assisted during the session. The obtained informed consent was recorded in the data file. Data derived from the study questionnaire were inserted into a custom-made database together with demographic and other clinical variables, in particular type of CIED (pacemaker [PM] or implantable cardioverter defibrillator [ICD]), MRI conditionality (yes/no), and date of implant.

The primary endpoint of our study was to verify how many MRI scans had been requested for our CIED-implanted patients since implant. The secondary endpoints were: number of MRIs examinations performed following MRI requests; body zones studied; incidence of requested MRI scans in PM and ICD patients; incidence of MRI scans requested according to device MRI conditionality (MRI conditional versus non-MRI conditional). The study was approved by the Independent Ethical Committee of IRCCS Istituto clinico Humanitas, and all patients’ information was de-identified.

### Statistical analysis

Statistical analyses were performed with Medcalc software (www.medcalc.net). Continuous variables are presented as median (interquartile range [IQR]). Comparison between categorical variables was performed using a Fisher’s exact test; comparison between continuous variables was performed using a Kruskal–Wallis test. For both tests, a p value lower than 0.05 was considered statistically significant.

## Results

During the 10-month study period, 795 patients were interviewed (508 male, 63.9%; median age 75 yrs., IQR 70–84 yrs.); 560 patients (70.4%) were implanted with a PM and 235 with an ICD (29.6%) **(Table 1)**. MRI conditional devices were implanted in 221 patients (27.8%; 133 PMs and 78 ICDs); leads and devices producers are described in Table 2. Median follow-up was 1678 days (IQR 1512–1792 days). These patients constitute our study group.

**Table 1:** MRI scan request.

	Total (n = 795)	MRI requested (n = 121)	MRI not requested (n = 674)	p
**Male, n (%)**	508 (64%)	79 (65%)	429 (64%)	NS
**Age, yrs**	75 ± 12	71 ± 13	76 ± 12	< 0.00001
**PM, n (%)**	550 (69%)	91 (75%)	469 (70%)	NS
**ICD, n (%)**	245 (31%)	30 (25%)	205 (30%)	NS
**MRI-conditional devices, n (%)**	221 (28%)	46 (38%)	175 (26%)	< 0.001
**Time from implant, days (median, 25th-75th percentile)**	1678 (1035–2674)	1828 (1194–2700)	1554 (956–2655)	0.059

yrs = years; PM = Pacemaker; ICD = Implantable cardioverter–defibrillator; MRI = Magnetic resonance imaging.

**Table 2:** Leads and device producers.

	Device, n (%)	Leads, n (%)
**Medtronic**	164 (20.6)	453 (32.2)
**Boston Scientific**	53 (6.7)	92 (6.5)
**MicroPort**	127 (16.0)	109 (7.7)
**Biotronik**	425 (53.4)	698 (49.5)
**Abbott**	26 (3.3)	57 (4.1)

Boston Scientific was formerly known as Guidant, MicroPort as Sorin or LivaNova and Abbott as StJude Medical. Leads and devices were grouped with last brand name.

**Fig 1:**
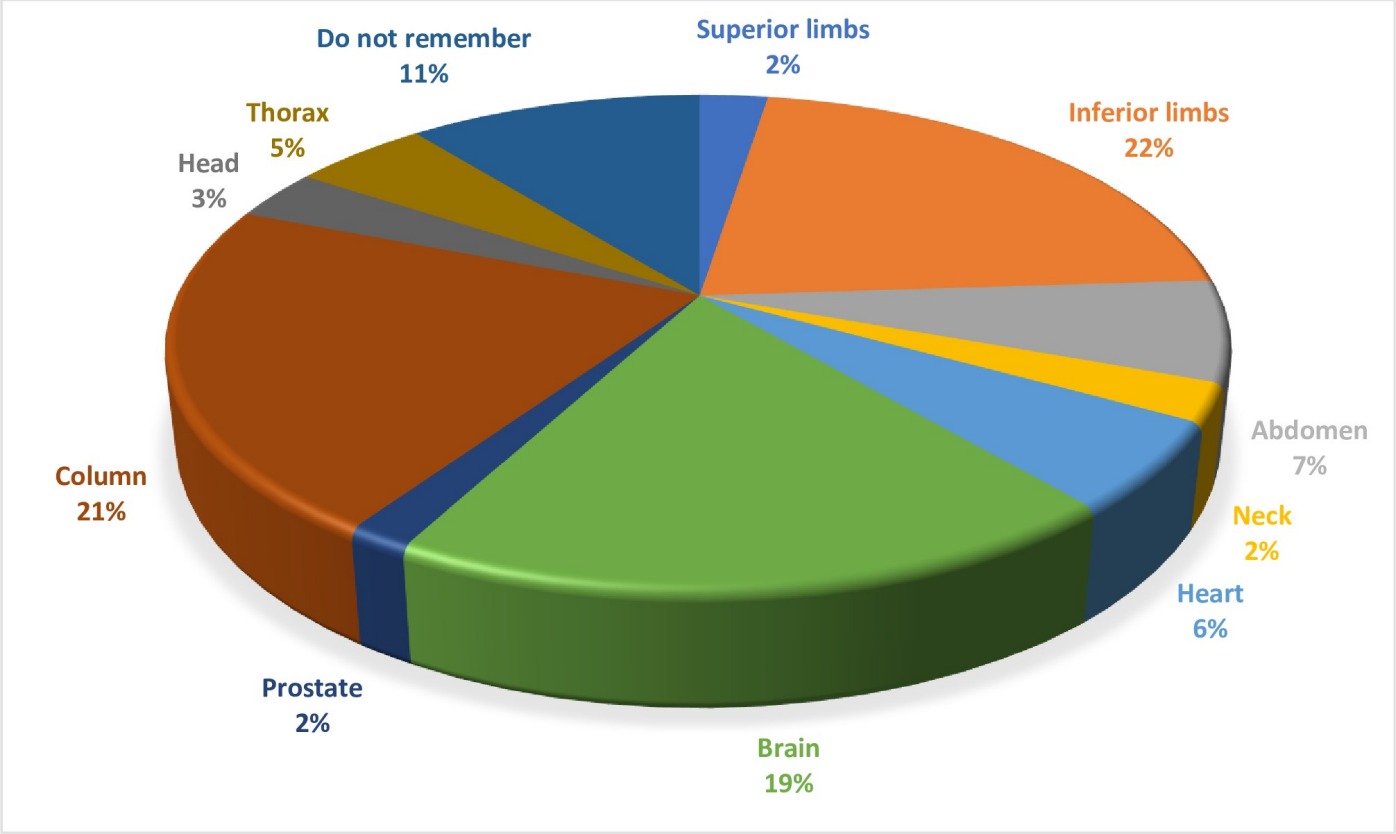
Sites of MRI scan request.

At the moment of interview, an MRI scan had been requested for 121 patients (15.2% of the whole population; 91 PMs and 30 ICDs, p = NS; 2.7/100 patient years). An MRI scan was requested in 16.4% of patients implanted with a PM and in 12.8% of patients implanted with an ICD (p = NS). An MRI scan was requested in significantly more patients with MRI conditional devices (n = 46 [20.8%]; 6.4/100 patient years) than in patients with non-MRI conditional devices (n = 75 [13.1%]; 2.0/100 patient years) (p = 0.008). Surprisingly, only 20 patients for whom an MRI was requested (15.2%), including one implanted with a non-MRI conditional device, actually had an MRI examination: none of them experienced problems in devices functioning. The body zones for which MRI was requested are shown in **Fig 1**. MRI was requested most often for the lower limbs (n = 26), vertebral column (n = 26) and brain (n = 23), and less often for the abdomen (n = 8), heart (n = 7), thorax (n = 6), head (n = 4), upper limbs (n = 4), neck (n = 3) and prostate (n = 2); 12 patients could not remember the body zone for which MRI had been requested **(Fig 1)**.

There was no difference in the number of MRI scans requested according to gender (14.6% of female patients and 15.5% of male patients, p = NS), but patients in whom the MRI was requested were younger (mean age 71 ± 13 yrs vs 76 ± 12 yrs, p <0.0001). Time from implant was significantly longer for patients in whom the MRI was requested, with a trend towards significance (p = 0.059). Even dividing patients into two groups, one including patients implanted from less than 6 years and the other with longer time from implant, the difference is not statistically significant (incidence of MRI scan requests 13.4% vs 16.9%; OR 1.27, 95% CI 0.86–1.87, p = 0.23).

An MRI conditional device (RR 1.46, 95% CI 1.13–1.90, p = 0.004), an age lower than 75 years old (RR 1.62, 95% CI 1.17–2.25, p = 0.03) and time since implant > median follow-up (1678 days) (RR 1.21, 95% CI 1.02–1.43, p = 0.027) were significantly associated with a request for an MRI scan, both in univariate and multivariate models (data of multivariate analysis are reported in **Table 3**).

**Table 3:** Analysis of covariance–D’Agostino-Pearson test.

	Coefficient	SE	T	P
**ICD**	- 0.4394	0.02954	-2.452	0.0144
**MRI conditional device**	0.05437	0.03048	1.784	0.0484
**Age < 75 yrs old**	0.08165	0.02988	2.732	0.0064

ICD = ICD = Implantable cardioverter–defibrillator.

## Discussion

Our study shows that an MRI scan was requested during follow-up in 15% of the real-world patients at our centre in Bergamo, Italy, implanted with a CIED. The rate is far lower than the 75% of patients needing an MRI scan reported by Kalin and coll. in 2005 [3]. A study published in 1999 [8] showed that 17% of patients implanted with a PM had a problem related to electromagnetic interference because of an MRI scan. Even more recent data, published by Taruya and coll. in 2015 [9], found that over a 38-month follow-up period, 16.7% of patients implanted with a PM at their centre received a request to have an MRI scan, with an increase in requests of 5.3% every year. Celentano and coll. [10] found a rate of MRI scans in patients implanted with an MRI conditional CIED or with a system in which at least one component (lead or device) was MRI conditional of 7.0/100 patient years. In our study, if we take into consideration only patients with an MRI conditional device, the rate is 6.4/100 patient years. This rate is much higher than that in patients without an MRI conditional device. These data seem to suggest that the presence of an MRI conditional encourages use of this imaging tool in a higher number of patients, or–by the other side–the absence of an MRI conditional device encourages the use of other imaging exams (other from MRI) since the very beginning of the patients diagnostic iter. Anyhow, despite this in our series only half of patients in whom an MRI was requested had an MRI examination. It is impossible to say if this difference between MRI requests and examinations is linked to lack of trust in the reliability of these devices or to the lack of a prespecified protocol (not all MRI scans were performed or requested at our hospital). The difference could also be due to the non-existence of a specific law on the use of MRI for these particular devices or to the recommendations made by scientific societies. The Heart Rhythm Society (HRS) [11], for instance, recommends (class IA) strict adherence to manufacturer manuals and the existence of a specific protocol designed by cardiologists and radiologists before performing MRI scans in CIED patients, even if the implanted CIED is MRI conditional.

The study by Celentano and coll. [10], which took into consideration all patients in whom at least one component of the system was MRI conditional, touches on an interesting aspect of MRI conditionality. A system is considered MRI conditional only if all the parts (leads and CIED) are MRI conditional and all of them come from the same producer. So called “hybrid systems” composed of MRI conditional components from different manufacturers are not MRI conditional because the system “in toto” has not been certified. Theoretically, there are no reasons why such a system should not be MRI conditional; if MRI can be performed safely in patients with non-MRI conditional devices then hybrid systems should cause no particular problems. In fact, the HRS recommendations on MRI in CIED patients (class IIa, level of evidence B) [11] state an MRI scan can be performed in patients without an MRI conditional device, provided that there are no fractured, epicardial or abandoned leads. Many studies have already demonstrated that MRI scans, even in patients without MRI conditional devices, are not linked to any particular harmful events [12–14], especially if a specific protocol has been designed by the centre conducting the MRI examinations [15,16].

If there were no difference in cost between MRI conditional and non-MRI conditional devices, there would be no reason to use non-MRI conditional devices. However, differences do exist and we are therefore obliged to consider economic as well as clinical aspects. In patients who undergo device replacement this is particularly important, considering that it is systems, not individual leads or devices, that are MRI conditional. The increase in expense entailed by system replacement is–in our opinion–unjustified. Lead extraction before an MRI scan should also be carefully evaluated, taking into consideration the risk of lead extraction versus the risk of MRI in a patient with a non-MRI conditional system.

### Limitations

Our study was retrospective in nature and the results rely on patients’ ability to remember MRI requests and examinations; furthermore, median follow-up is 1678 day (almost 3 years) and therefore probably not long enough to confirm the data we found; anyway even in patients with a follow-up longer than 5 years, incidence of MRI scan–request does not increase in a statistically significant way in comparison to patients with shorter follow-up. Even if we cannot exclude that real incidence would be higher than that found in our study (for example for patients who died from cancer during the time past since previous follow-up), nevertheless, the study enrolled a sizeable number of patients, all followed-up at a single centre, from both an electrophysiological and radiological point of view.

## Conclusions

The incidence of MRI scan requests in our series of real-world CIED-implanted patients was 15%. The incidence was higher amongst patients with MRI conditional devices, but not all patients for whom a scan was requested actually had an MRI exam. In our study, incidence of MRI scan requests increases with time from implant, but not in a statistically significant way.

## Supporting information

S1 Data (XLSX)
